# Supplementary material for: Impact of Change in Body Composition during Follow-Up on the Survival of GEP-NET
Source: Cancers (Basel). 2022 Oct 22;14(21):5189. doi: 10.3390/cancers14215189 (PMC9654293; doi:10.3390/cancers14215189)
Supplement: Supplementary file 1 [file cancers-14-05189-s001.zip › Suplementary material S1.pdf]

FEATURED PROTOCOL FROM NIH IMAGEJ SOFTWARE TO MEASURE BODY COMPOSITION AT L3 LEVEL IN COMPUTED TOMOGRAPHY

Already published protocol just tuned to be easier:

- DOI: 10.1002/jpen.1721
- https://doi.org/10.1371/journal.pone.0211629

Steps order is essential to semiautomatic protocol.

0. Automatically adjust brightness and contrast

Image → Adjust → Brightness and contrast → Auto (emerging window)

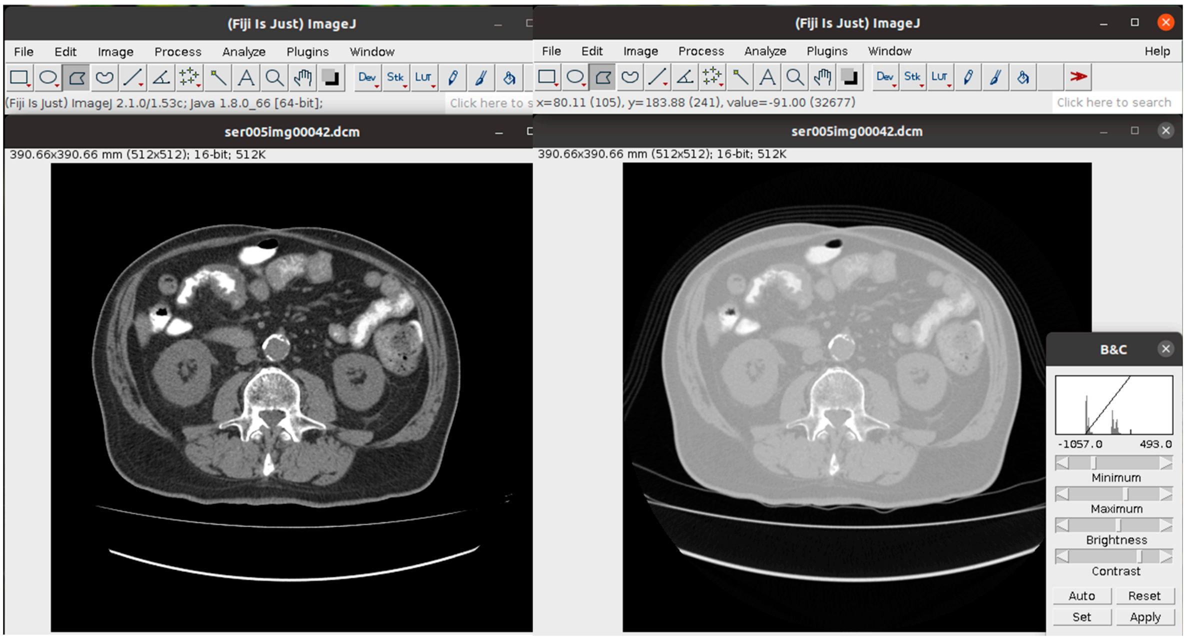

1. Define body outline

We define a body outline setting a general threshold (-250, 1000) to fill the section in red. Then, we use the "Wand" tool to click inside. Automatically, the software marks the edge in yellow. Finally, we measure.

<u>STEP 1:</u>

Image → Adjust → Threshold → Set (emerging window)

STEP 2:

(emerging window)
Lower threshold level: -250
Upper threshold level: 1000

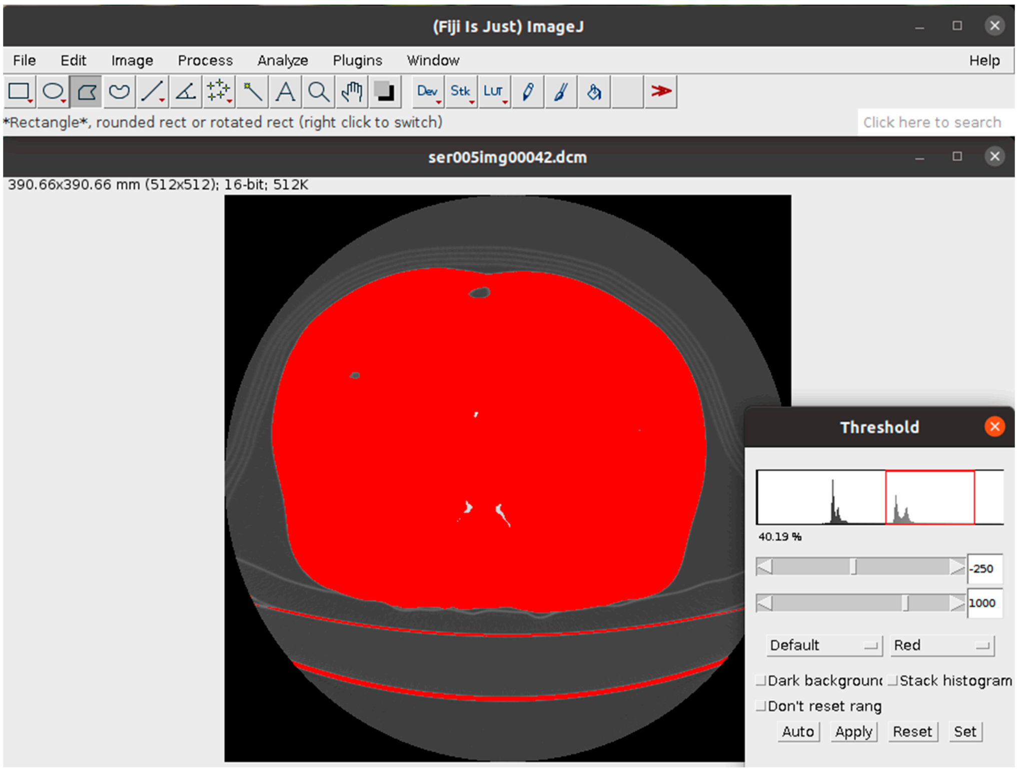

STEP 4:

Select the "Wand" tool and click inside the section.

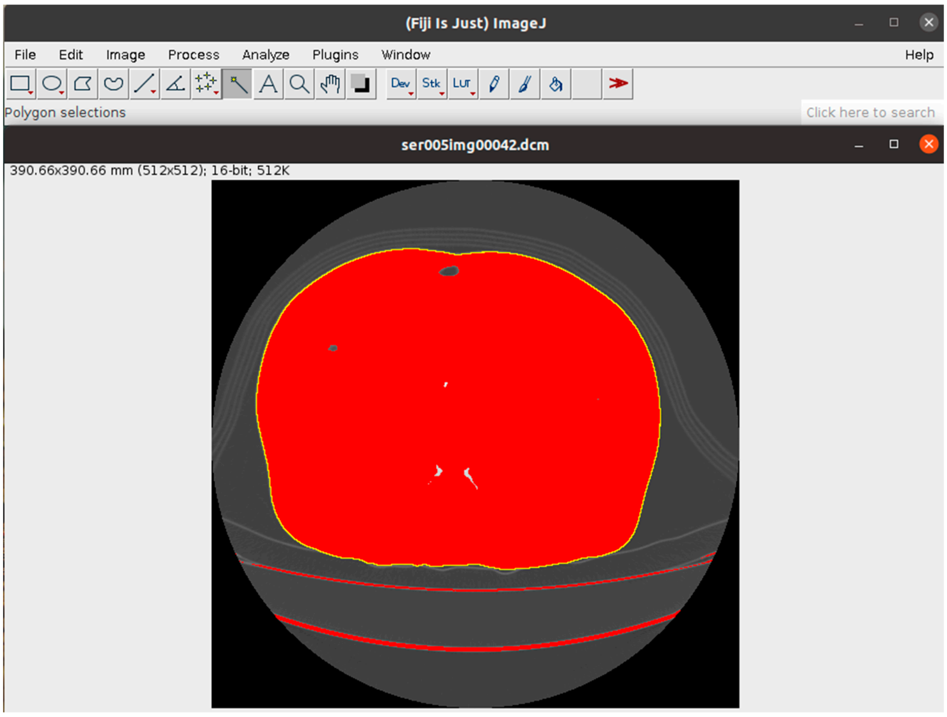

STEP 5:

Analyze → *Set measurements* → Select *Area* and *Perimeter* (emerging window)

It is important to unmark “Limit to threshold” in this phase.

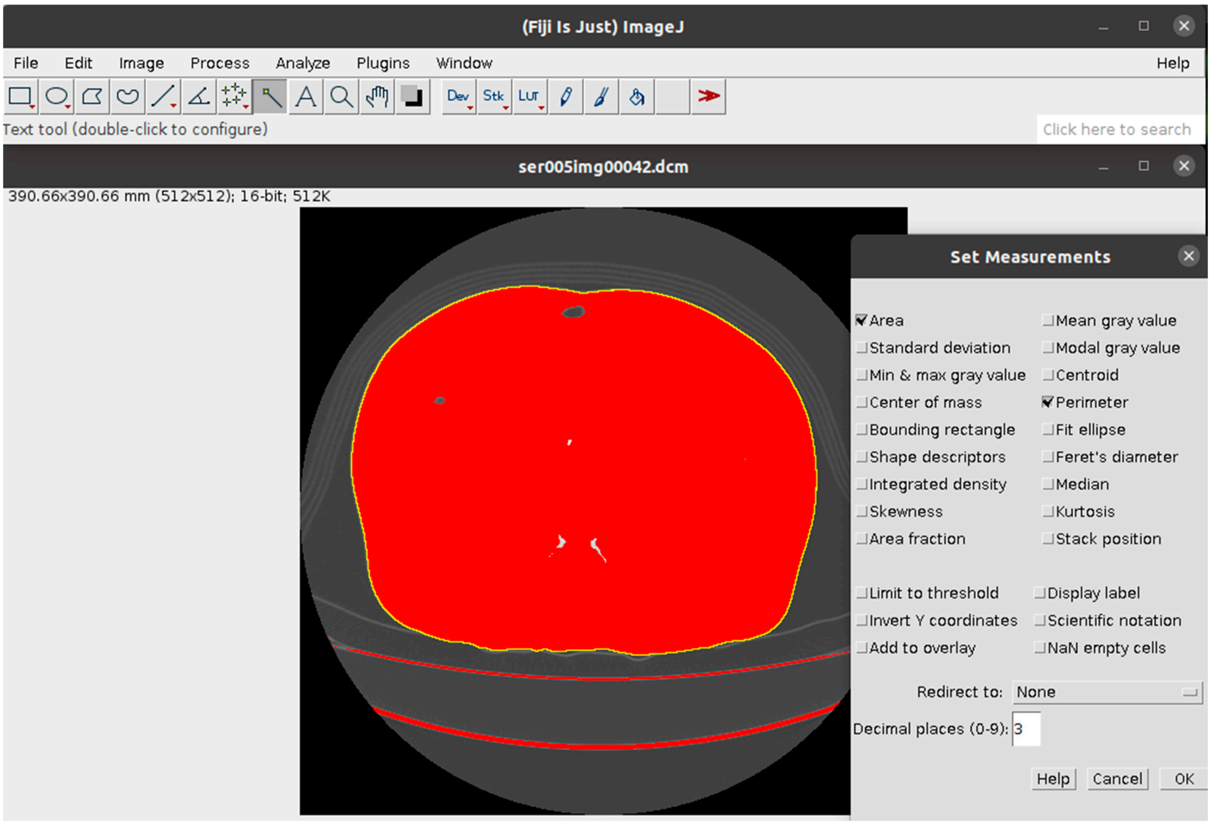

STEP 6:

Analyze → Measure

It will appear an emerging window with the result as in the example. We must not close it to save them at the end of the protocol.

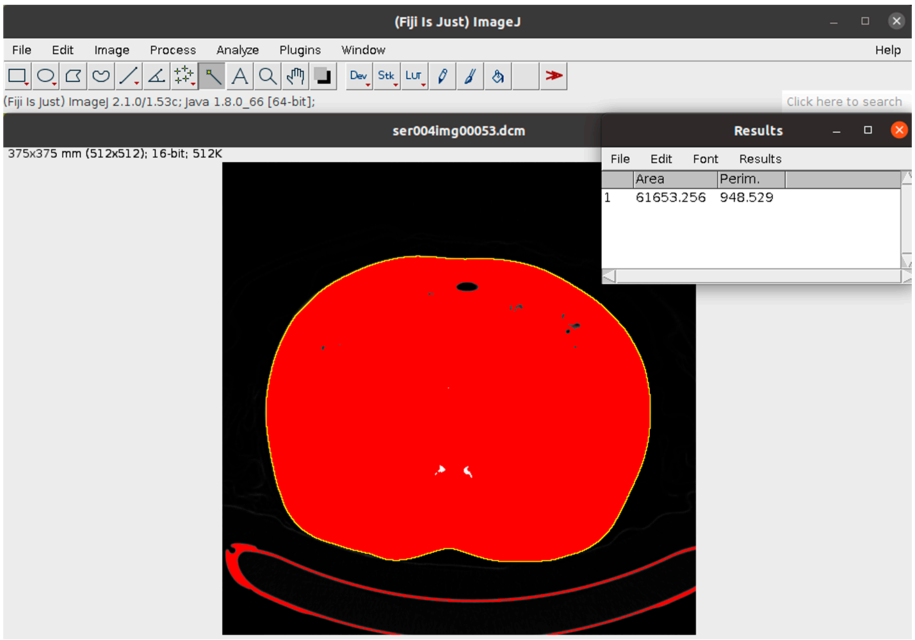

STEP 7:

We measure fat setting its Hounsfield units:

Image → Threshold → Set (ventana emergente)

Lower threshold level: -190
Upper threshold level: -30

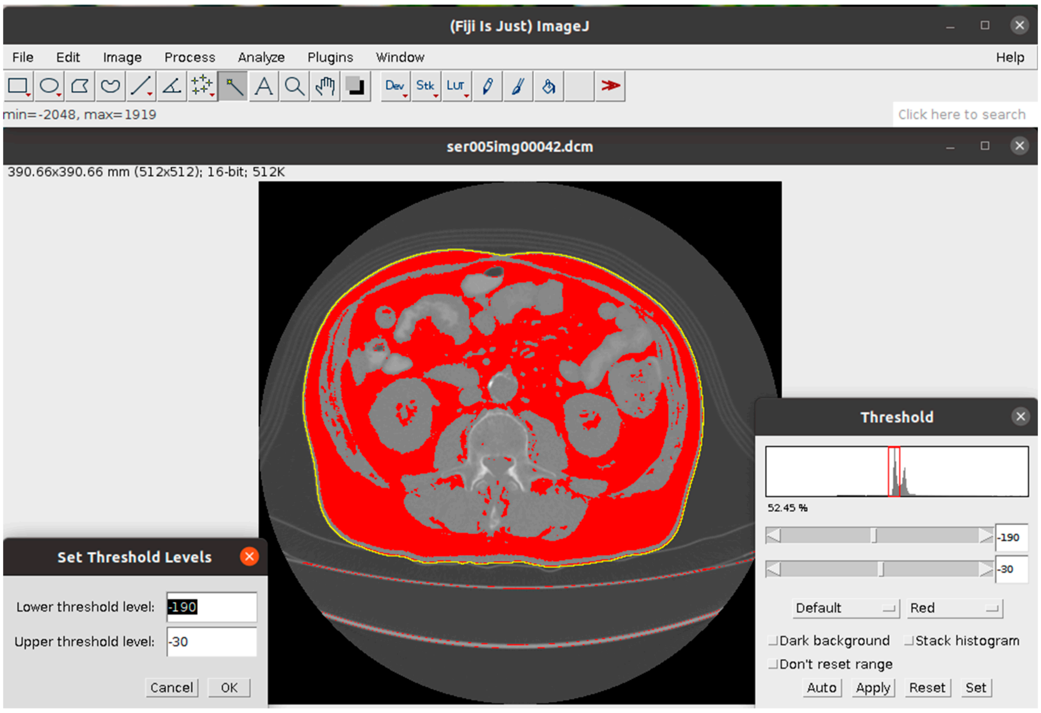

STEP 8:

Since this point, we have to change "Set measures" marking "Limit to threshold". It takes into account the Hounsfield units we establish. Areas will appear in red.

Analyze → *Set measurements* → Select *Area*, *Perimeter* and *Limit to threshold* (emerging window)

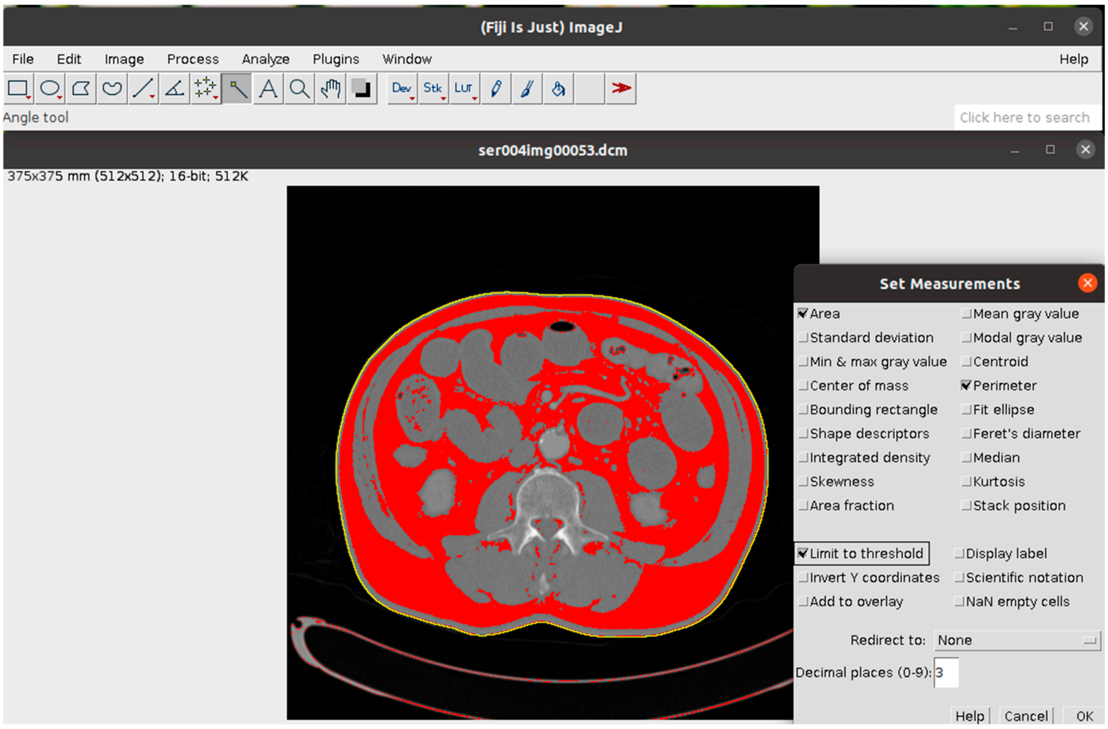

STEP 9:

We measure total fat: subcutaneous, visceral, intermuscular and the error inside the guts. All of them share Hounsfield units and they are inside the yellow line that establishes the limit. The current measure will be added to the Results window.

Analyze → Measure

2. Delimit muscle outline perimeter

STEP 1:

Hounsfield units from -250 to -250 for a more comfortable vision.

Image → Threshold → Set (emerging window)

Lower threshold level: -250
Upper threshold level: -250

STEP 2:
We use the “Polygon selections” tool to delimit the perimeter by clicking as in the image below.

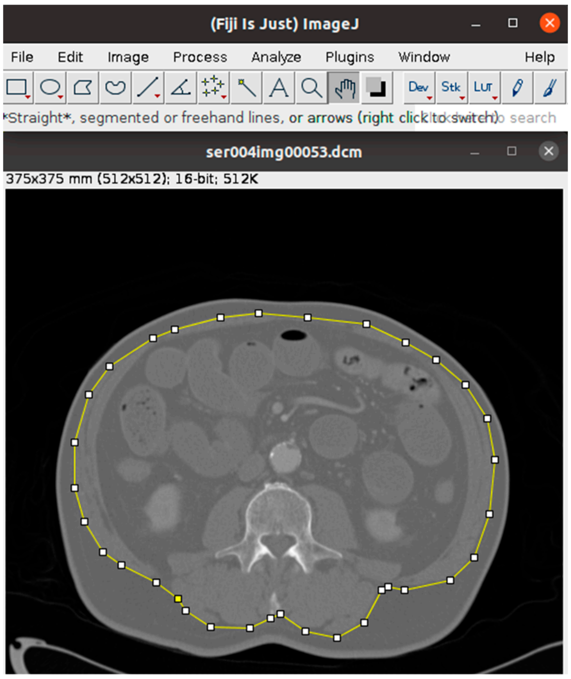

STEP 3:

Plugins → Macros → Run

Automatic: We run *BC_ImageJ_1.imj* macro which sets all the Hounsfield units in terms of guessing different density muscle and fat. It will be important to subtract the areas. Measures will appear in the Results window below the previous ones.

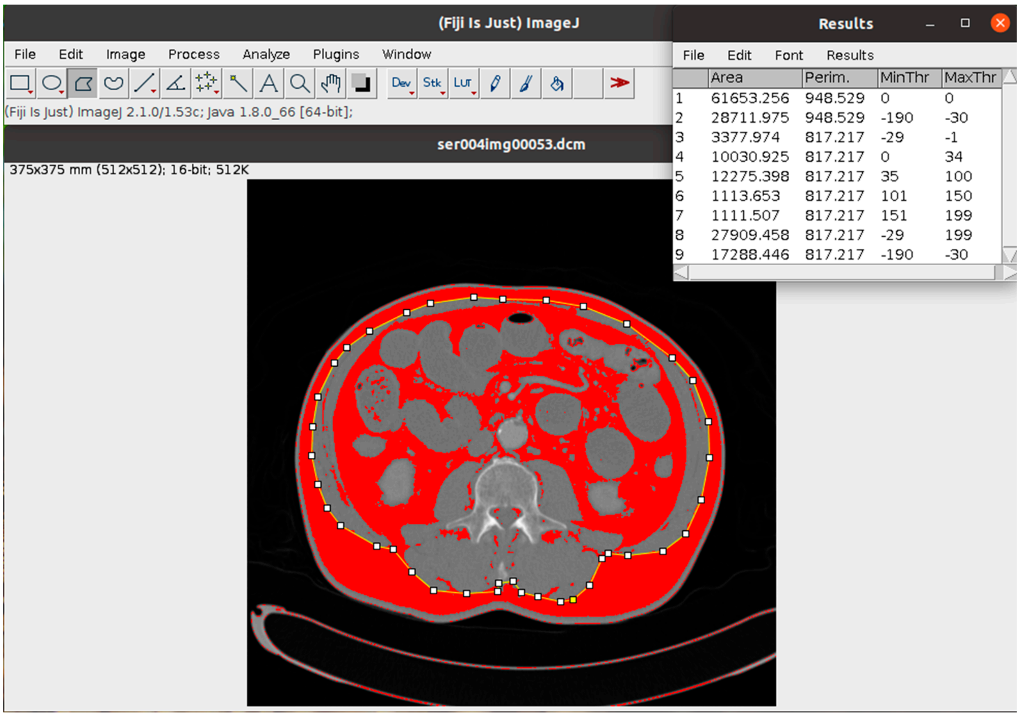

3. Delimit muscle inner line

<u>STEP 1:</u>

Hounsfield units from -250 to -250 for a more comfortable vision.

Image → *Threshold* → *Set* (emerging window)

Lower threshold level: -250
Upper threshold level: -250

<u>STEP 2:</u>

We use the “Polygon selections” tool to delimit the perimeter by clicking as in the image below.

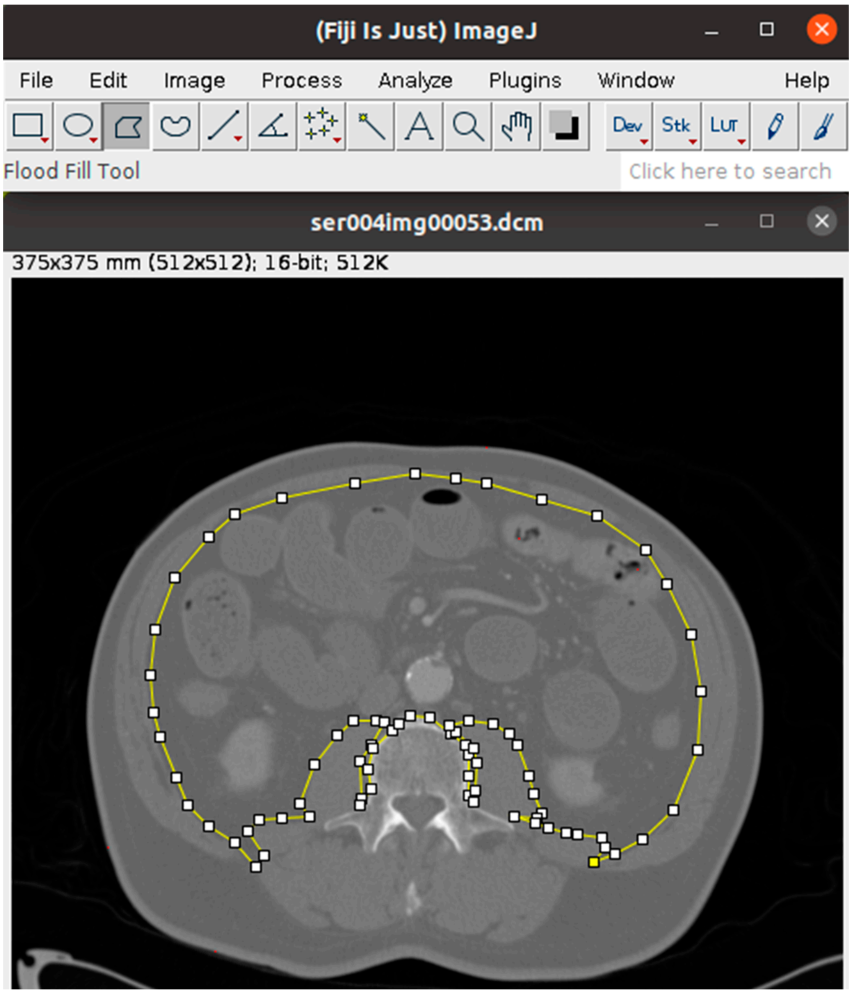

STEP 3:

Plugins → Macro → Run

Automatic: We run *BC_ImageJ_1.imj* macro which sets all the Hounsfield units in terms of guessing different density muscle and fat. It will be important to subtract the areas. Measures will appear in the Results window below the previous ones.

4. Delimit vertebra perimeter

STEP 1:

Hounsfield units from -250 to -250 for a more comfortable vision.

Image → Threshold → Set (emerging window)

Lower threshold level: -250
Upper threshold level: -250

STEP 2:

We use the “Polygon selections” tool to delimit the vertebra perimeter by clicking as in the image below.

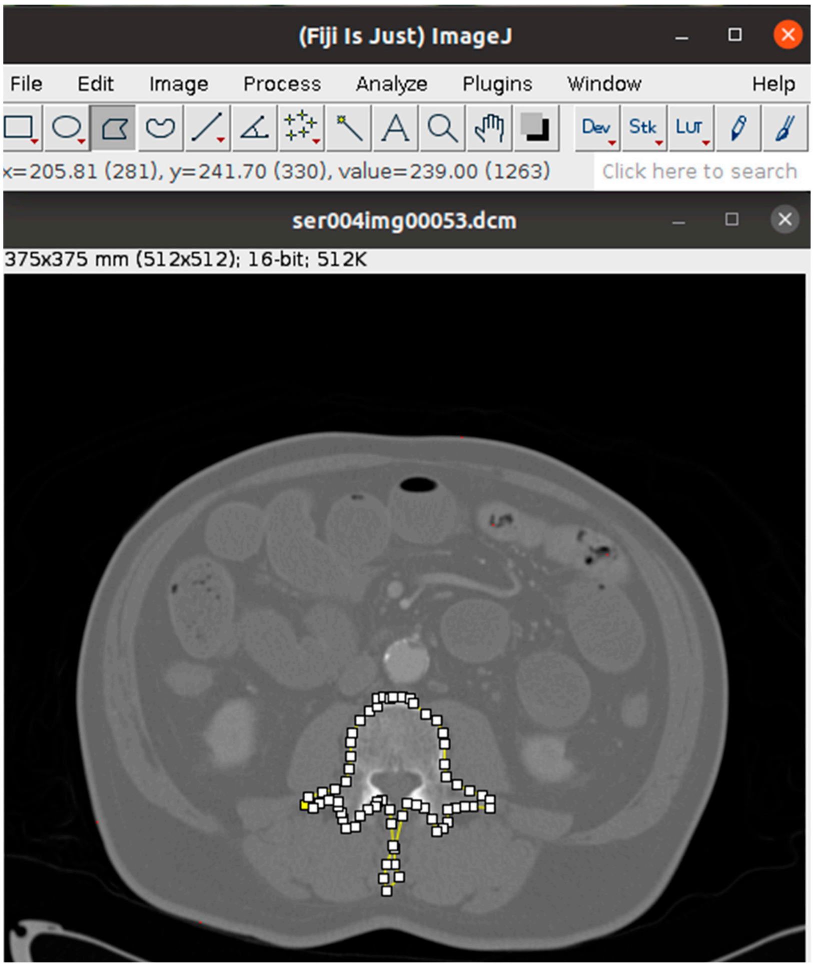

STEP 3:

Plugins → Macro → Run

Automatic: We run *BC_ImageJ_1.imj* macro which sets all the Hounsfield units in terms of obtaining possible zones that share Hounsfield units with muscle and fat in the vertebra area. It will be important to subtract the areas. Measures will appear in the Results window below the previous ones.

5. Delimit zones inside the guts that share Hounsfield units with fat

STEP 1:

Hounsfield units from -250 to -250 for a more comfortable vision.

Image → *Threshold* → *Set* (emerging window)

Lower threshold level: -190
Upper threshold level: -30

<u>STEP 2:</u>

We can use “Wand” or “Polygon sections” tools being helped by “Shift” to keep the selections.

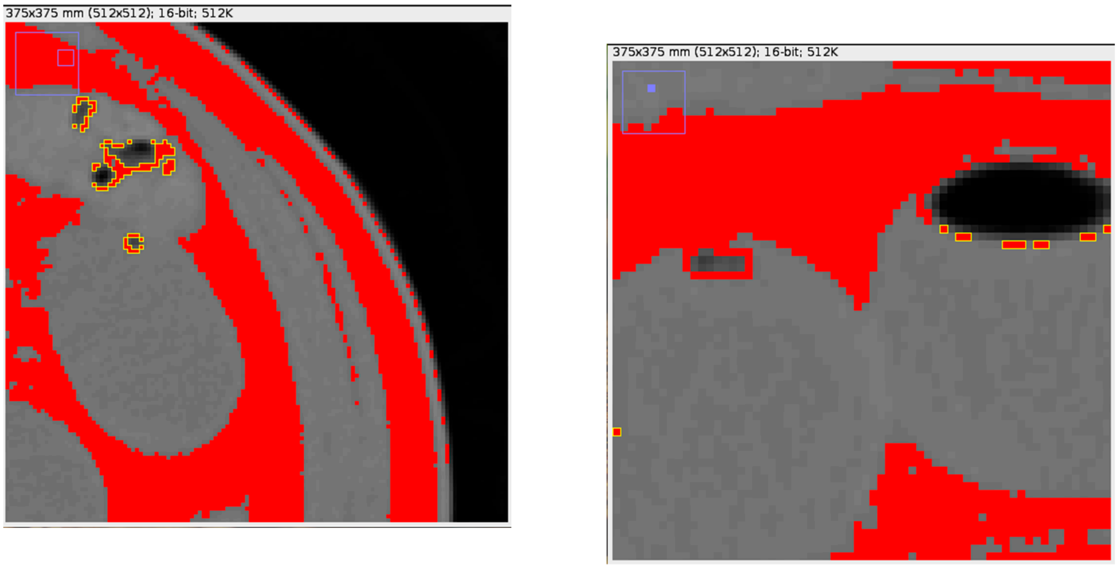

Using “Wand”

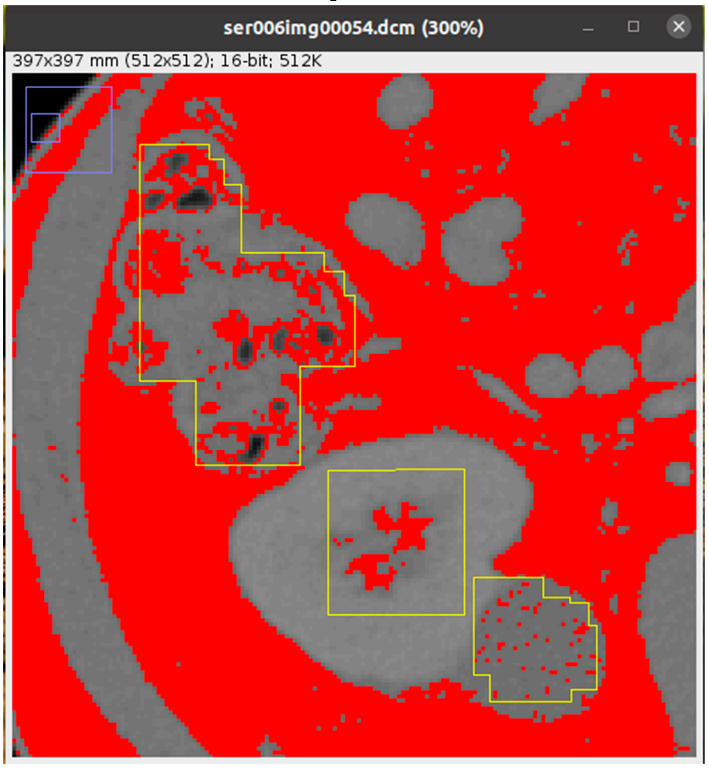

Using “Polygon selection”

STEP 3:

We measure the area of these selected zones

Analyze → Measure

6. We save all the results in a .csv file

Results emerging window → *Files* → *Save as*
The order and number of measures (24) have to be accurate to run R code.
