# Supplementary material for: Impact of Change in Body Composition during Follow-Up on the Survival of GEP-NET
Source: Cancers (Basel). 2022 Oct 22;14(21):5189. doi: 10.3390/cancers14215189 (PMC9654293; doi:10.3390/cancers14215189)
Supplement: Supplementary file 1 [file cancers-14-05189-s001.zip › Suplementary material S2.pdf]

Correlations of somatostatin analogues and body composition.

Measure (cm²/m²)	Overall	Not somatostatin analogues	Somatostatin analogues	p value	Test
**Total body area**	262.085 (64.196)	270.093 (68.595)	245.569 (51.099)	0.0508	T-test
**Subcutaneous fat area**	66.918 (32.882)	69.68 (35.727)	61.223 (25.631)	0.3899	Mann-Whitney wilcoxon
**Visceral fat area**	61.346 (38.857)	66.928 (39.165)	49.834 (36.127)	0.0168	Mann-Whitney wilcoxon
**Intermuscular fat area**	5.579 (3.825)	6.1 (4.181)	4.505 (2.714)	0.0272	Mann-Whitney wilcoxon
**Total fat area**	133.844 (59.289)	142.709 (60.824)	115.562 (52.245)	0.0254	T-test
**Very low density muscle area**	4.864 (2.74)	5.207 (2.903)	4.156 (2.249)	0.0567	Mann-Whitney wilcoxon
**Low density muscle area**	13.225 (5.331)	13.674 (5.335)	12.297 (5.285)	0.2324	T-test
**Normal density muscle area**	23.759 (8.002)	23.206 (7.526)	24.899 (8.922)	0.359	T-test
**Total muscle area**	42.36 (7.561)	42.626 (7.326)	41.812 (8.117)	0.6327	T-test

Correlations of metformin and body composition.

Measure (cm²/m²)	Overall	Not metformin	Metformin	p value	Test
**Total body area**	262.085 (64.196)	252.89 (60.527)	298.124 (60.589)	0.0107	T-test
**Subcutaneous fat area**	66.918 (32.882)	67.224 (33.545)	72.703 (36.294)	0.5762	T-test
**Visceral fat area**	61.346 (38.857)	53.603 (31.685)	81.591 (45.552)	0.0319	Mann- Whitney wilcoxon
**Intermuscular fat area**	5.579 (3.825)	5.432 (4.052)	5.796 (3.042)	0.4404	Mann- Whitney wilcoxon
**Total fat area**	133.844 (59.289)	126.26 (56.53)	160.089 (59.544)	0.0444	T-test
**Very low density muscle area**	4.864 (2.74)	4.575 (2.518)	5.697 (3.217)	0.2085	Mann- Whitney wilcoxon
**Low density muscle area**	13.225 (5.331)	12.478 (4.772)	15.922 (5.675)	0.0305	T-test

**Normal density muscle area**	23.759 (8.002)	24.386 (8.135)	20.896 (7.603)	0.1058	T-test
**Total muscle area**	42.36 (7.561)	41.979 (7.467)	42.924 (8.761)	0.5191	Mann- Whitney wilcoxon

Correlations of body composition and T2DM.

**Measures (cm^2/m^2)**	**Overall**	**No T2DM**	**T2DM**	**p value**	**Test**
**Total body area**	262.05 (64.24)	251.7895 (62.82453)	275.5544 (59.36912)	0.07663	T-test
**Subcutaneous fat area**	66.59 (33.12)	70.44305 (34.47183)	62.95904 (32.01727)	0.1497	Mann- Whitney wilcoxon
**Visceral fat area**	61.55 (38.72)	50.72197 (31.29403)	72.84834 (39.37159)	0.01505	Mann- Whitney Wilcoxon
**Intermuscular fat area**	133.76 (59.36)	5.230239 (4.155015)	5.928339 (3.094747)	0.1719	Mann- Whitney Wilcoxon
**Total fat area**	5.61 (3.82)	126.3953 (57.78653)	141.7357 (56.92119)	0.3159	Mann- Whitney Wilcoxon
**Very low density muscle area**	4.88 (2.73)	4.342222 (2.437471)	5.509409 (2.857705)	0.06624	Mann- Whitney Wilcoxon
**Low density muscle area**	13.27 (5.29)	12.16497 (4.95841)	14.59226 (4.924284)	0.06267	Mann- Whitney Wilcoxon
**Normal density muscle area**	23.67 (8.02)	24.82547 (8.381614)	21.66515 (6.909492)	0.05621	T-test
**Total muscle area**	42.33 (7.59)	41.87893 (7.905753)	42.22696 (7.21791)	0.45	Mann- Whitney Wilcoxon

Type 2 diabetes mellitus and body composition.

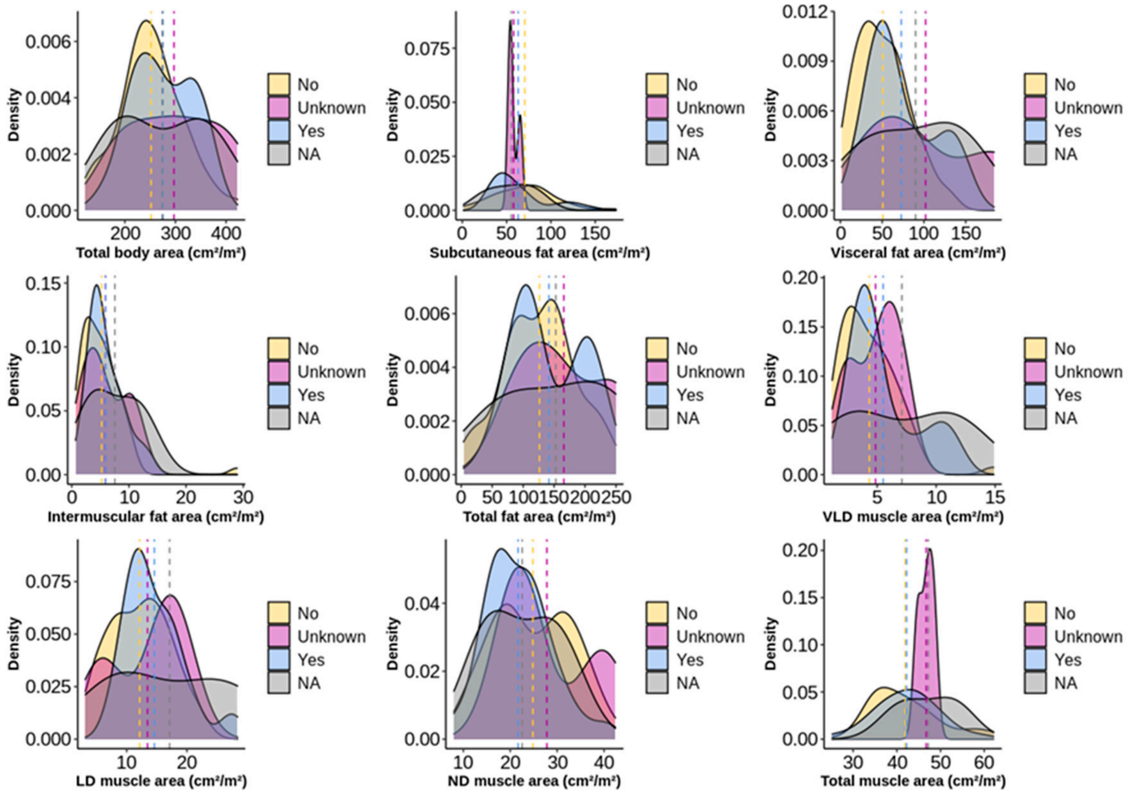

Distribution plots of body composition according to diagnosis of Type 2 Diabetes Mellitus.
